# Coherent interfaces govern direct transformation from graphite to diamond

**DOI:** 10.1038/s41586-022-04863-2

**Published:** 2022-07-06

**Authors:** Kun Luo, Bing Liu, Wentao Hu, Xiao Dong, Yanbin Wang, Quan Huang, Yufei Gao, Lei Sun, Zhisheng Zhao, Yingju Wu, Yang Zhang, Mengdong Ma, Xiang-Feng Zhou, Julong He, Dongli Yu, Zhongyuan Liu, Bo Xu, Yongjun Tian

**Affiliations:** 1 Center for High Pressure Science (CHiPS), State Key Laboratory of Metastable Materials Science and Technology, Yanshan University, Qinhuangdao, China; 2 Key Laboratory for Microstructural Material Physics of Hebei Province, School of Science, Yanshan University, Qinhuangdao, China; 3 School of Physics and MOE Key Laboratory of Weak-Light Nonlinear Photonics, Nankai University, Tianjin, China; 4 Center for Advanced Radiation Sources, The University of Chicago, Chicago, IL USA; 5 School of Materials and Chemical Engineering, Zhongyuan University of Technology, Zhengzhou, China

**Keywords:** Composites, Phase transitions and critical phenomena, Atomistic models, Surfaces, interfaces and thin films

## Abstract

Understanding the direct transformation from graphite to diamond has been a long-standing challenge with great scientific and practical importance. Previously proposed transformation mechanisms^1–3^, based on traditional experimental observations that lacked atomistic resolution, cannot account for the complex nanostructures occurring at graphite−diamond interfaces during the transformation^4,5^. Here we report the identification of coherent graphite−diamond interfaces, which consist of four basic structural motifs, in partially transformed graphite samples recovered from static compression, using high-angle annular dark-field scanning transmission electron microscopy. These observations provide insight into possible pathways of the transformation. Theoretical calculations confirm that transformation through these coherent interfaces is energetically favoured compared with those through other paths previously proposed^1–3^. The graphite-to-diamond transformation is governed by the formation of nanoscale coherent interfaces (diamond nucleation), which, under static compression, advance to consume the remaining graphite (diamond growth). These results may also shed light on transformation mechanisms of other carbon materials and boron nitride under different synthetic conditions.

## Abstract (web summary)

The discovery of graphite–diamond hybrid carbon, Gradia, which consists of graphite and diamond nanodomains interlocked through coherent interfaces, clarifies the long-standing mystery of how graphite turns into diamond.

## Main

Carbon has numerous allotropes owing to its ability to form various bonds through orbital hybridization. Among all the allotropes, graphite and diamond (with *sp*^2^ and *sp*^3^ hybridization, respectively) are the most ubiquitous and have been extensively exploited by humans for several millennia. Although both occur in nature, the synthesis of diamond from graphite was not successful until the middle of the last century^6,7^. The transformation from graphite to diamond can be made under different synthetic conditions, such as high pressure, high temperature (HPHT) with^6^ or without^7,8^ a catalyst, explosive shock^9^, and low-temperature compression under severe shear deformation^10^. Along with these experimental efforts, understanding the transformation from graphite to diamond has attracted broad attention but remained a significant challenge^11^.

Largely based on diffraction data from recovered samples, several concerted transformation mechanisms were proposed to account for the graphite-to-diamond transformation^1,2^. In hexagonal graphite (HG), graphene layers are arranged in AB-type stacking, with carbon atoms in each layer bonded covalently in a honeycomb-like lattice through *sp*^2^ hybridization. According to the concerted transformation mechanisms, HG undergoes several possible variations in stacking order to transform into cubic diamond (CD) or hexagonal diamond (HD) where all carbon atoms are bonded covalently by *sp*^3^ hybridization. The AB stacking may change into ABC stacking, followed by collective puckering to transform into CD^2^. Alternatively, the AB stacking may change either to AA stacking followed by puckering to transform into HD^1^, or to AB′ stacking followed by puckering to transform into CD or buckling to transform into HD^2^. Some reports, again largely based on diffraction data, have suggested that formation of HD is energetically favoured at lower synthesis temperatures^12^. This prompted nucleation-and-growth models^3,13^ with two types of transient heterophase junction proposed between diamond nuclei and the graphite matrix^11,14^: one is a graphite–diamond diphase connected with weak van der Waals interaction, and the other is covalently bonded interfaces between diamond and graphitic domains with a reduced interlayer distance of less than 2.5 Å. Similar to the nucleation-and-growth mechanisms, a wave-like lattice buckling and slipping model suggested a stacking-order change from AB to ABC by bending graphitic layers, followed by formation of transient heterophase junctions to complete the transformation to CD^15^.

Despite the numerous mechanisms proposed, the graphite-to-diamond transformation process remains elusive. The main obstacle to understanding the transformation is that the process occurs under HPHT without in situ information, particularly at the atomic scale. Post-mortem examinations on the structure of products recovered from HPHT-treated graphite typically rely on X-ray diffraction (XRD), which is insensitive to small amounts of defects or intermediate phases in the sample. In the absence of microscopic information, interpretation of the XRD data is sometimes non-unique, thus leading to different conclusions^12,16,17^. More recently, high-resolution transmission electron microscopy (HRTEM) has been applied to natural and laboratory-shocked samples^4,5^, and has revealed two types of diamond–graphene composite nanostructure, which are named as type 1 and type 2 diaphite structures following the original definition of diaphite^18^. In type 1 diaphite, a few graphene layers are inserted parallelly within {111} diamond; in type 2 diaphite, graphitic layers are inserted at high angles within {113} diamond^4,5^. The proposed crystal structure gives rise to diffraction peaks resembling those of graphite (with an interlayer spacing of 3.0 Å) and CD. Although the origin of this hybrid structure and its correlation with the graphite-to-diamond transformation remains unclear^4,5^, the idea of a hybrid structure provides an alternative view of the reported ‘compressed graphite’ with a 3.1-Å interlayer spacing^12,19–23^, and may play an important role in understanding the graphite-to-diamond transformation.

In this study, we investigate the products from graphite treated under static HPHT conditions with state-of-the-art scanning transmission electron microscopy (STEM). Partially transformed samples are characterized by graphite and diamond nanodomains interlocked via coherent interfaces. The graphite domains, with interlayer spacings centring at about 3.1 Å, are intimately connected to diamond domains with numerous stacking faults. Atomic-resolution high-angle annular dark-field (HAADF) STEM observations reveal four basic structural motifs constituting the graphite–diamond interfaces. Theoretical calculations suggest a progressive graphite-to-diamond transformation process characterized by formation of graphite–diamond interfaces and subsequent advance of the interfaces for diamond growth, consistent with the atomically resolved interface structures as well as interface propagation observed by in situ STEM. This work thus clarifies the long-standing puzzle since the first successful static synthesis of diamond.

**Fig. 1:**
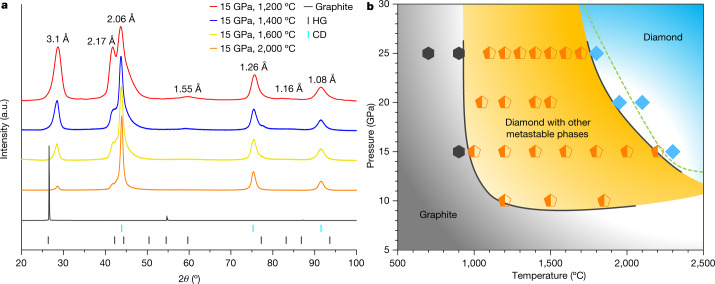
XRD patterns and phase evolution diagram of graphite under HPHT. **a**, XRD of samples recovered from 15 GPa and 1,200 °C, 1,400 °C, 1,600 °C and 2,000 °C. The pristine graphite is included for comparison. The coloured tags at the bottom indicate standard diffraction lines of graphite (HG) and cubic diamond (CD). **b**, Kinetic phase diagram of graphite under HPHT determined from the XRD results. Hexagons, pentagons and diamond symbols represent samples that are pure graphite, mixed phases containing CD and other metastable carbon phases, and pure diamond, respectively. Collectively, these data points define three regions as delineated by the solid lines. The dashed line is the established phase boundary between graphite and diamond.

Selected XRD patterns of partially transformed samples recovered from 15 GPa and temperatures between 1,200 °C and 2,000 °C are shown in Fig. 1a, along with the pristine graphite whose strong and sharp (00*l*) peak indicates excellent crystallinity. After HPHT treatment, the main diffraction peaks are consistent with those previously observed in graphite compressed at moderate temperatures^12^, where peaks not belonging to CD were attributed to the so-called compressed graphite (3.1 Å and 1.55 Å) and HD (2.17 Å and 1.16 Å). Such assignments, however, are under debate^12,24^. With increasing synthesis temperature and under identical heating duration, intensities of diffraction peaks from CD increase, whereas the other peaks gradually diminish. A kinetic phase diagram is constructed based on XRD measurements, as shown in Fig. 1b. Graphite remains unchanged in low-temperature (*T* < 900 °C) and low-pressure (*P* < 10 GPa) regions. Above 900 °C and 10 GPa, a multiphase region emerges (orange field), where CD co-exists with other metastable carbon phases such as compressed graphite. At sufficiently high temperatures and pressures, the recovered samples are predominantly CD (light blue field). The well established equilibrium phase boundary between graphite and diamond is drawn as the dashed line^25^.

**Fig. 2:**
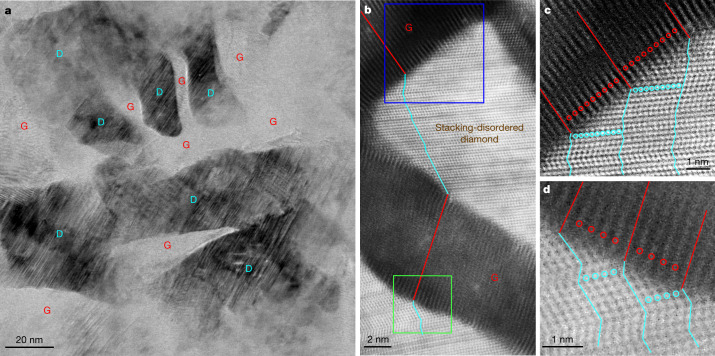
Microstructures of a sample recovered from 15 GPa and 1,200 °C. **a**, Low-magnification BF-STEM image showing nanoscaled diamond (D) domains embedded in graphite (G). **b**, High-resolution HAADF-STEM image of graphite domains showing a reduced interlayer spacing of 3.1 Å and diamond domains with numerous stacking faults, with well defined interfaces between the two phases. Alternating red and cyan lines delineate the end-to-end connectivity between one atomic layer in graphite and kinked carbon bilayer in diamond traversing multiple graphite and diamond domains. **c**, **d**, Magnified HAADF-STEM images corresponding to the blue-boxed (**c**) and green-boxed (**d**) regions in **b**. The red and cyan lines and circles highlight the one-to-one correspondence between the atomic layers in graphite and the kinked carbon bilayers in diamond, respectively.

**Extended Data Fig. 1:**
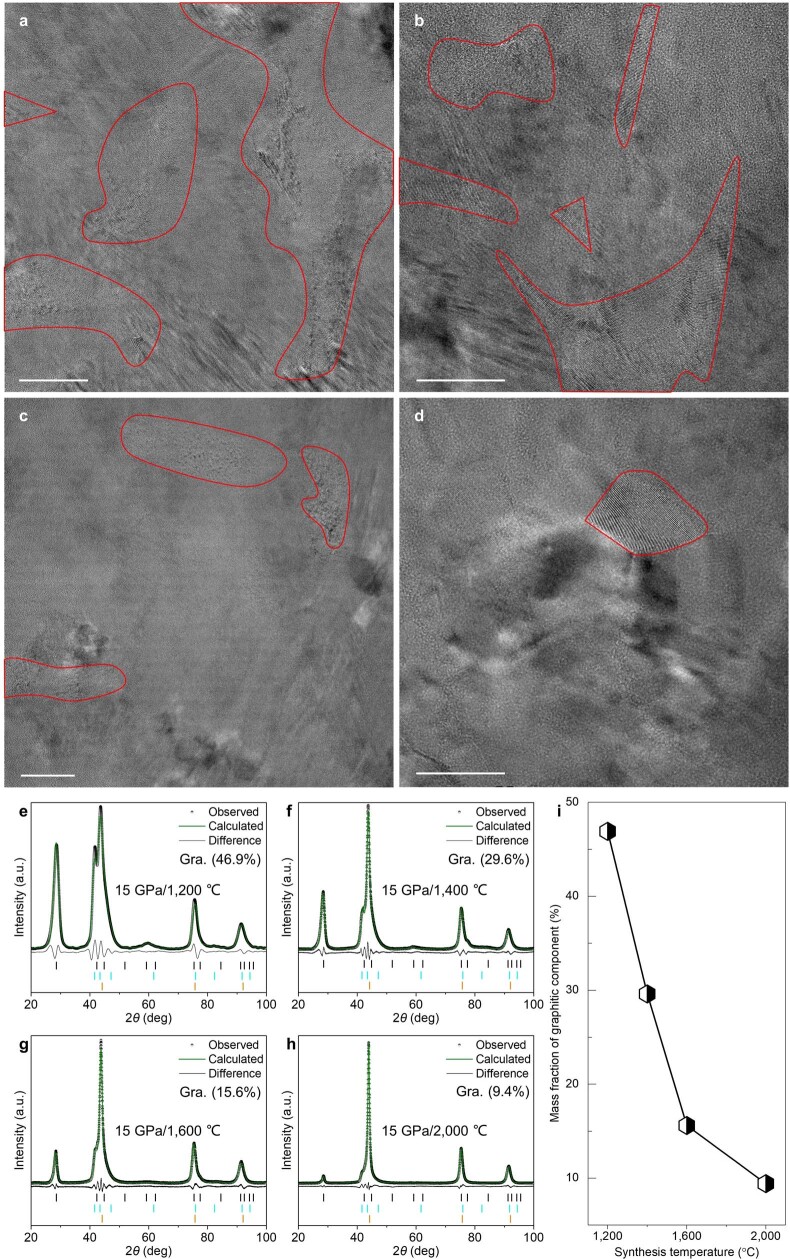
Typical microstructures and Rietveld refinement analysis of samples recovered from 15 GPa and various temperatures. **a**, 1,200 °C. **b**, 1,400 °C. **c**, 1,600 °C. **d**, 2,000 °C. With increasing synthesis temperature, the graphitic regions (enclosed with red curves) in the recovered samples decreases gradually. All scale bars are 20 nm. **e−h**, Rietveld refinement for different samples. Black bars: 'compressed graphite'; cyan bars: HD; orange bars: CD. **i**, Mass fraction of graphitic component in the samples with increasing synthesis temperature.

Detailed TEM observations on quenched samples provide direct insight into the mechanism of graphite-to-diamond transformation under static compression. Extended Data Fig. 1a–d shows typical microstructures of samples recovered from 15 GPa and various temperatures. All recovered samples are composed of diamond and (compressed) graphite, and the fraction of the graphitic phase decreases with increasing synthesis temperature, which is consistent with the results from XRD and Rietveld refinement analysis (Fig. 1a and Extended Data Fig. 1e–i). Figure 2a is a bright-field (BF)-STEM image from a sample recovered from 15 GPa and 1,200 °C, in which diamond (D) and graphite (G) nanodomains are clearly distinguished. In neighbouring diamond and graphite domains, the lattice fringes of the two phases are tilted relative to one another, forming interfaces different from the (113)_CD_ or (111)_CD_ types as previously proposed for meteoritic or laboratory-shocked diamonds based on TEM observations^4,5,11,14,26^. High-resolution HAADF-STEM observations further confirm the tightly bonded graphitic and diamond domains (Fig. 2b). The graphite domains show a reduced interlayer spacing of about 3.1 Å, and the lattice fringes are distorted, especially adjacent to the interfaces. The diamond domains exhibit considerable stacking disorder in the close-packed carbon bilayers. Magnified HAADF-STEM images in Fig. 2c,d reveal a remarkable one-to-one correspondence between atomic layers in graphite and kinked carbon bilayers in diamond. Hereafter, this unique hybrid carbon, which consists of nanoscale graphite and diamond units bonding each other through coherent interfaces, is referred to as Gradia. The corresponding interface is referred to as the gradia interface. The phase/microstructure evolution of graphite under different pressure–temperature conditions (Fig. 1b and Extended Data Fig. 1) and the observed gradia interfaces suggest that the formation and migration of the interfaces play a decisive role in graphite-to-diamond transformation under static pressure: diamond growth is accomplished by advancing the interfaces into graphite.

**Fig. 3:**
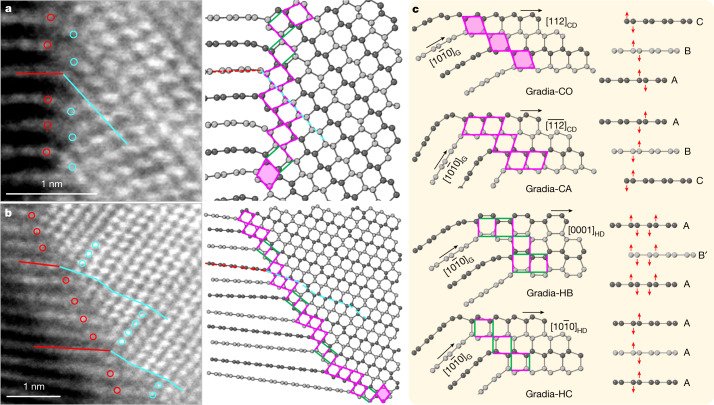
Coherent interface structures between graphite and diamond. **a**, **b**, Atomic-resolution HAADF-STEM images of two gradia interfaces (left) and the corresponding atomic models (right). The red and cyan lines (circles) delineate the one-to-one correspondence between graphite and diamond. In the atomic models, adjacent graphitic layers are coloured with different greyscales for clarity. Structural motifs at the interface are denoted with rhombi (with or without shadows) and rectangles (with different orientations). **c**, Four representative gradia interfaces. The pink and green sides in patterns indicate side lengths of 2.18 Å and 2.06 Å, respectively. See main text for details of nomenclature.

**Extended Data Fig. 2:**
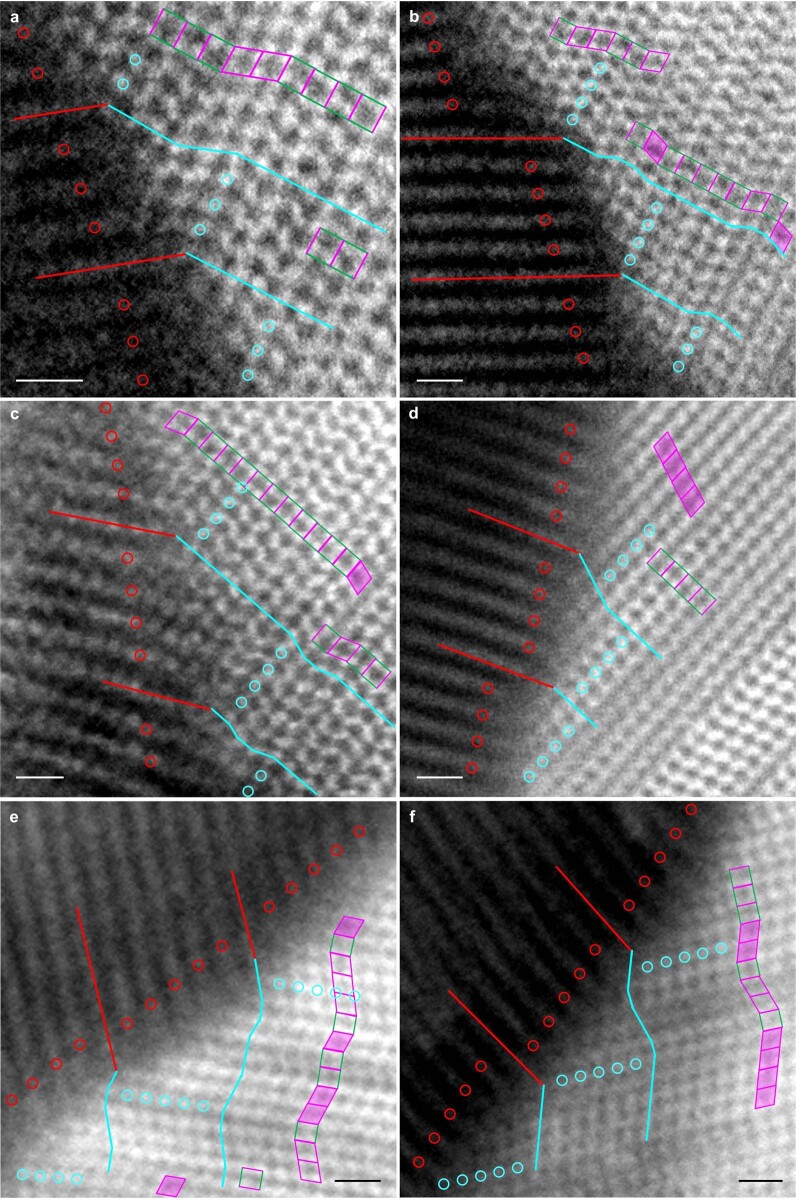
Atomic-resolution HAADF-STEM images of various gradia interfaces. **a**–**f**, The average interlayer distances in graphite regions are 3.13 Å (**a**), 3.11 Å (**b**), 3.20 Å (**c**), 3.02 Å (**d**), 2.93 Å (**e**), and 3.09 Å (**f**). Red and cyan lines/circles indicate the one-to-one coherence between the atomic layers in graphite and the kinked carbon bilayers in diamond. Different stacking modes in diamond regions are emphasized with rhombi and rectangles (CD and HD motifs), respectively. All scale bars are 0.5 nm.

Examples of HAADF-STEM images of gradia interfaces are shown in Fig. 3 and Extended Data Fig. 2. The graphite and diamond domains exhibit the following orientation relations: [1$\bar{2}$10]_G_//[1$\bar{1}$0]_CD_ or [1$\bar{2}$10]_HD_, with no definitive epitaxial relationship across the interface. On the basis of the HAADF-STEM observations, four primary structural motifs are identified to constitute the gradia interfaces, as shown in Fig. 3c where the corresponding puckering and buckling processes in graphitic layers with different stacking orders are indicated by red arrows. When viewed along [1$\bar{1}$0]_CD_, the (111)_CD_ and (11$\bar{1}$)_CD_ planes form a rhombic pattern with equal side lengths of 2.18 Å. A rhombus in CD can connect to the (0001) lattice of compressed graphite through a vertex with either an obtuse or an acute angle, forming two structural motifs, which are referred to as Gradia-CO and Gradia-CA, respectively. Similarly, when viewed along [1$\bar{2}$10]_HD_, the (10$\bar{1}$0)_HD_ and (0002)_HD_ planes form a rectangular pattern with two side lengths of 2.18 Å and 2.06 Å, respectively. The adjacent (0001) layers of compressed graphite can either buckle into a boat conformation and transform into (10$\bar{1}$0)_HD_ with a *d*-spacing (the distance between planes of atoms that give rise to the diffraction peaks) of 2.18 Å, or pucker into a chair conformation and transform into (0002)_HD_ with a *d*-spacing of 2.06 Å. These two structural motifs are referred to as Gradia-HB and Gradia-HC, respectively.

**Extended Data Fig. 3:**
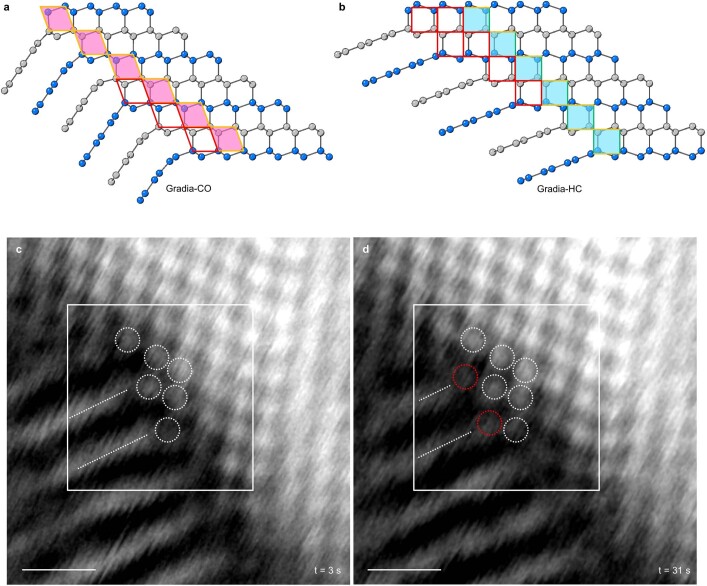
The advance of gradia interfaces into graphite. **a**, Several new Gradia-CO motifs (open red rhombi) advancing into graphite one step further from initial Gradia-CO interface (filled magenta rhombi) shown in Fig. 3c. **b**, Several new Gradia-HC motifs (open red rectangles) advancing into graphite one or more steps further from initial Gradia-HC interface (filled cyan rectangles) shown in Fig. 3c. **c**, **d**, The interface propagation with extra bonding under electron-beam irradiation during STEM observation. The LAADF-STEM images captured at the same area illustrate atomic bonding at the interface. The white dotted circles mark the C−C dumbbell units in diamond, and the red ones correspond to the newly formed C−C units in diamond at the interface. All scale bars are 0.5 nm.

**Extended Data Fig. 4:**
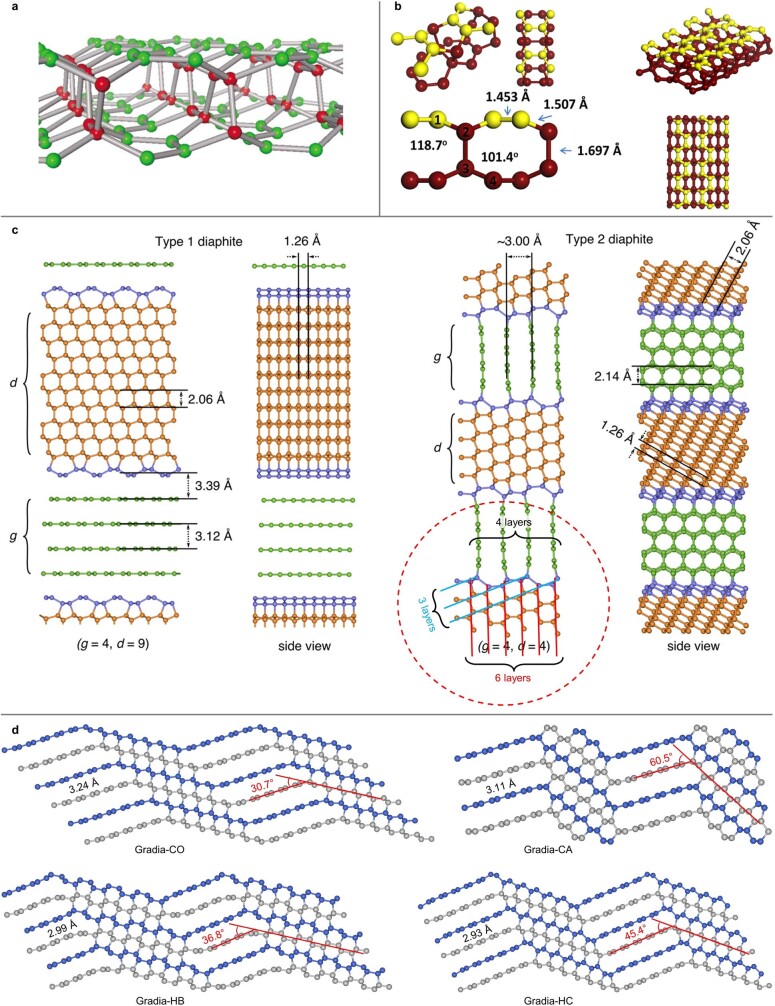
Comparison of different carbon structures. **a**, The originally defined diaphite, that is, a bilayer structure with one third of atoms (coloured in red) forming bonds between layers, which is referred to as '2D diaphite' hereafter, has neither graphite unit nor diamond unit in the structure (adapted with permission from ref. ^45^, Fig. 5c. Copyright 2009 by the American Physical Society). **b**, The originally defined 2D diaphite structure used for surface adsorption propertie calculation, yellow carbon atoms indicate extruded sites for hydrogen adsorption (adapted with permission from ref. ^46^, Fig. 1. Copyright 2017 by Elsevier B.V.). **c**, Model structures for type 1 and type 2 diaphite. For type 2 diaphite, graphite and diamond regions are also connected via bonded interfaces, but without a one-to-one correspondence: depending on the choice of diamond {111} planes, 4 graphene layers are corresponding to 6 or 3 diamond (111) planes (see the interface in the red dashed circle in **c**) (adapted from ref. ^5^, Fig. 3a and 3b. CC BY 4.0 (https://creativecommons.org/licenses/by/4.0/)). **d**, Four structural models for Gradia-CO, Gradia-CA^47^, Gradia-HB, and Gradia-HC with fully coherent interfaces, that is a one-to-one correspondence in atomic positions between graphite and diamond layers across the interface. They show different tilting angles between graphite and diamond layers and different interlayer spacings in graphitic regions. These newly found structures are clearly different from the 2D diaphite^45^, type 1, and type 2 diaphite^5^.

Under HPHT, atomic layers in graphite endure compression, bending and interlayer sliding, resulting in highly localized variations in interlayer distance, curvature and stacking order, which may induce new bonding across neighbouring graphite layers to form different interface structures. The gradia interface made up of the aforementioned structural motifs has great variability and flexibility to accommodate such local structural variations (Fig. 3c). It is noted that although both Gradia-HB and Gradia-HC can co-exist with Gradia-CO and Gradia-CA, Gradia-HB and Gradia-HC are mutually exclusive (Fig. 3 and Extended Data Fig. 2). This is because a plane cannot be completely filled by two differently oriented rectangles, with all vertices overlapping. Under HPHT conditions, gradia interfaces advance into graphite, promoting diamond growth. For example, Extended Data Fig. 3a,b shows schematically the advance of the Gradia-CO and Gradia-HC interfaces (Fig. 3c) into graphite, with several new motifs forming at the frontline. Similar growth processes also occur for other gradia interfaces with different combinations of structural motifs. As the interface advances to the graphite side, the specifically combined structural motifs impose constraints on the bonding of carbon atoms in adjacent graphite layers, resulting in significant stacking disorder of carbon bilayers in the as-grown diamond (Fig. 3 and Extended Data Fig. 2). The absence of a definitive epitaxial relationship across the gradia interface is also determined by such transformation processes. Instead, varying tilting angles between graphite and diamond layers across the interface as well as varying interlayer spacing between graphite layers are shown (Extended Data Fig. 2). Our Gradia structures are clearly different from previously proposed structures, such as type 2 diaphite^4,5,27^, and the interstratified graphite and diamond^26^, where definitive topotactic relationships were observed between graphite and diamond (Extended Data Fig. 4). It may be worth noting that the STEM observations did not identify any pure HD domains in the recovered samples, even though the XRD patterns show a prominent peak at 2.17 Å and two weaker ones at 1.93 Å (shoulder) and 1.16 Å, which were previously attributed to HD^12^. Actually, all diamond domains are characterized by a high density of stacking faults. Similar hexagonal-cubic stacking disorders also exist in natural and laboratory-shocked diamonds^24^, and account for the hexagonal feature in diffraction patterns^16,17^. One thus should exercise caution when claiming new diamond phases. This potential ambiguity does not preclude the existence of HD though. For example, we did observe an HD nanodomain, 3 nm in thickness and 30 nm laterally from HPHT-treated carbon onions^28^. Larger HD phases may be produced with carefully selected carbon precursors and fine-tuned pressure–temperature conditions.

**Fig. 4:**
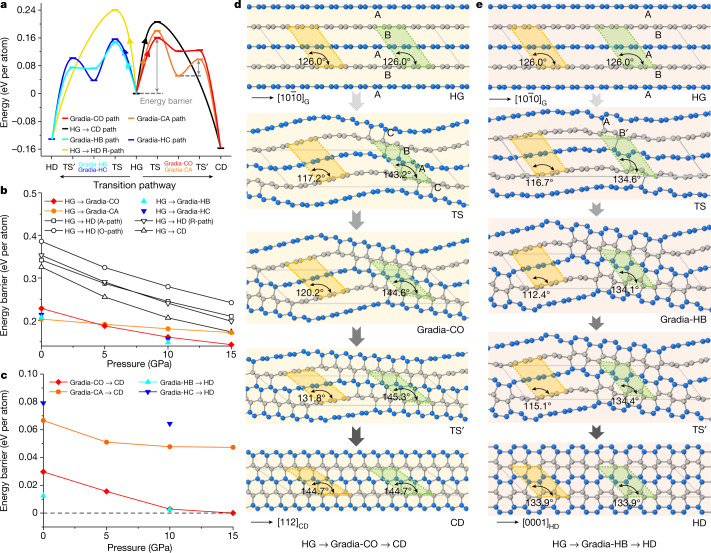
Energy barriers and transformation process from graphite to diamond through intermediate crystals (hypothetic) containing gradia interfaces. **a**, Energy profile of graphite-to-diamond transformation through different pathways at 10 GPa. The maximum energy barrier occurs when the wavy graphitic layers start bonding, that is, forming the gradia interface along HG to intermediate crystal. TS represents the transition states of the pathway from graphite to intermediate crystal; TS’ represents the transition states of the pathway from intermediate crystal to diamond. **b**, Energy barriers decrease with increasing pressure from HG to intermediate crystal. The classic concerted transformation pathways previously proposed—that is, HG→CD and HG→HD (A-path, R-path, O-path)—are from ref. ^29^. **c**, Energy barriers decrease with increasing pressure from intermediate crystal to CD (or HD). The energy barrier in this stage (diamond growth) is significantly lower than that in the nucleation stage (gradia interface formation). Above 10 GPa, Gradia-CO and Gradia-HB crystals can convert into diamond with almost no energy barrier. **d**,**e**, The structure snapshots during graphite-to-diamond transformation through Gradia-CO (**d**) and Gradia-HB crystals (**e**) at 10 GPa. The adjacent graphitic layers are distinguished with grey and blue colours. Under pressure, the graphite layers bend wavily. Bonding across graphite layers starts in the green-shadowed regions with a reduced interlayer spacing of about 2.1 Å, whereas the interlayer spacing increases from 2.9 Å to 3.2 Å in the yellow-shadowed regions, leaving graphite stable. Next, the interfaces advance gradually into graphite, and diamond nuclei eventually grow into pure diamond. The angles in the green- and yellow-shadowed regions indicate the localized changes in the structure.

**Extended Data Fig. 5:**
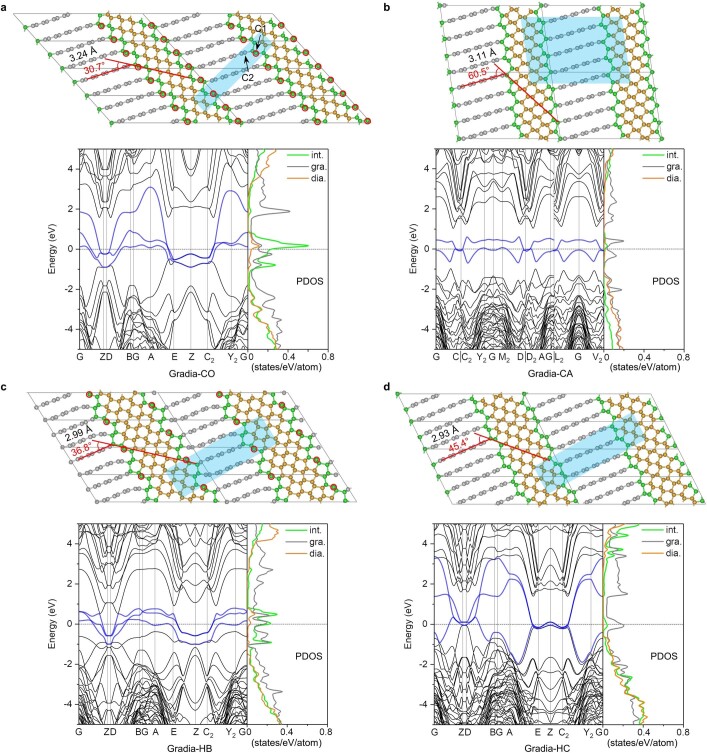
Hypothetical crystal structures with characteristic gradia interfaces and the corresponding electronic structures. **a**, Gradia-CO. **b**, Gradia-CA. **c**, Gradia-HB. **d**, Gradia-HC. The hypothetical crystals are named after the gradia interfaces shown in Fig. 3c. For each of the crystal models displayed, the thickness of the cell perpendicular to the page is the lattice constant of graphite [010]_HG_ (or [110]_CD_ and [010]_HD_). Details of structural information are listed in Extended Data Table 1. In each crystal structure, the grey-line grid indicates the unit cell employed for transformation energy barrier calculation, the cyan-shadowed area indicates the primitive cell. Carbon atoms are differently coloured: grey for *sp*^2^-hybridized atoms in graphitic sections, gold for *sp*^3^-hybridized atoms in diamond-structured sections, and green for those in gradia interfaces (among the interface atoms, atoms circled in red are *sp*^2^-hybridized, the others are *sp*^3^-hybridized). The lower part of each panel shows the calculated electronic structure with the bands across the Fermi level coloured in blue. In each DOS graph, the partial DOS (in unit of states per eV per atom) from interface, graphitic and diamond-structured atoms are coloured in green (int.), grey (gra.) and gold (dia.), respectively. Substantial contributions from the interface atoms to the density of states (DOS) near the Fermi level are revealed, especially for Gradia-CO and Gradia-HB crystals (**a** and **c**) with *sp*^2^-hybridized atoms at the interface. For instance, the electronic structure and partial density of states (PDOS) of Gradia-CO demonstrates a clear metallicity because of several electron bands across the Fermi level. The electronic states around Fermi level mainly come from the *p*_x_ and *p*_z_ orbitals from two sets of carbon atoms: C1 atoms (*sp*^2^-hybridized ones at the interface, red-circled) and C2 atoms (at the zigzag edge of graphene layer connecting the interface).

**Extended Data Fig. 6:**
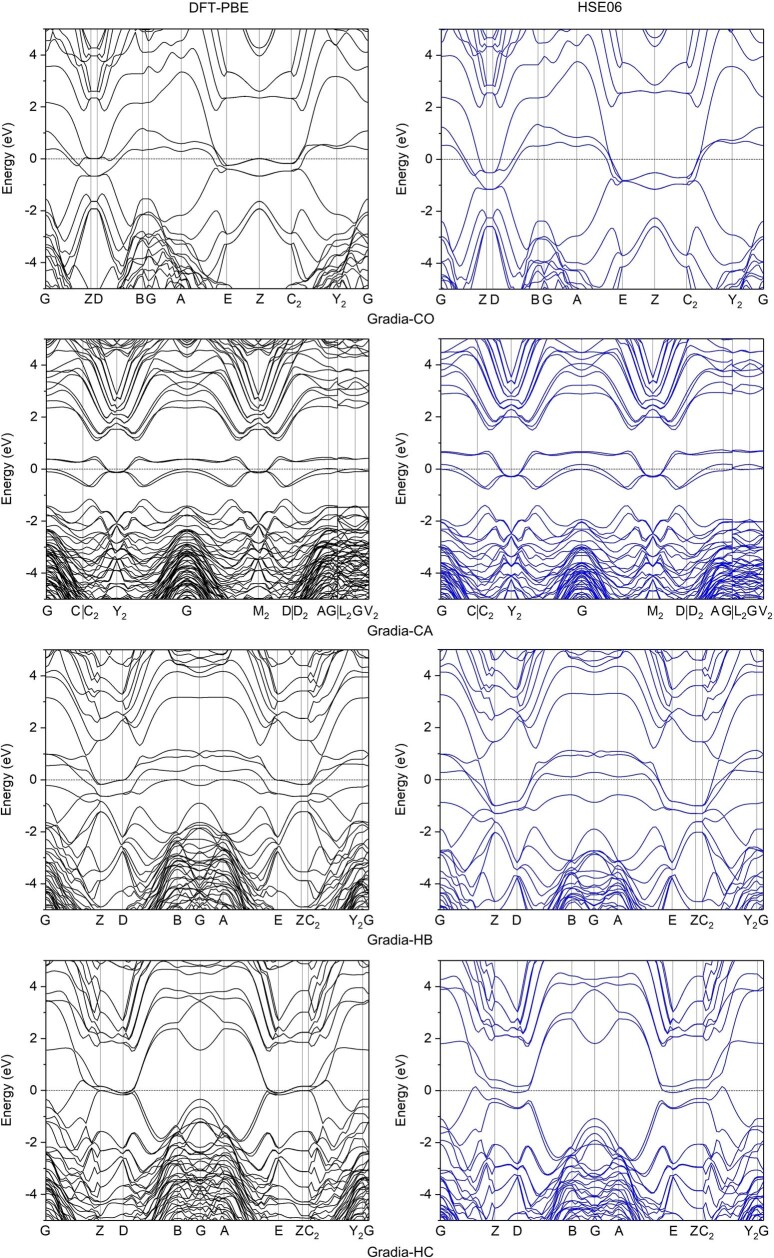
The ambient-pressure band structures of four Gradia structures calculated by DFT-PBE and HSE06 functional as implemented in VASP code. There are bands across the Fermi level, indicating metallic Gradia-CO, Gradia-CA, Gradia-HB, and semimetallic Gradia-HC at ambient pressure.

**Extended Data Fig. 7:**
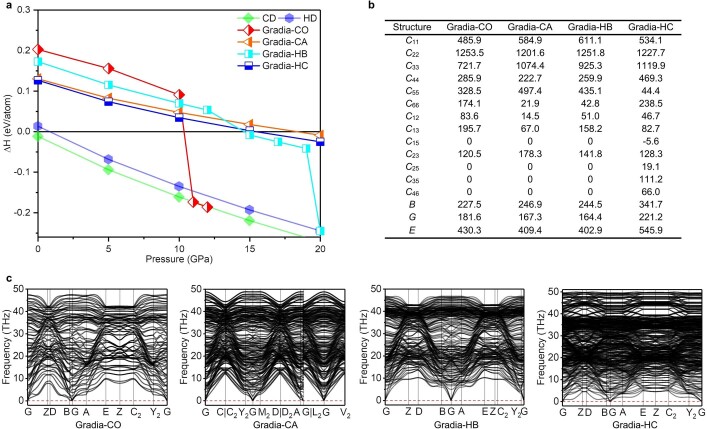
Thermodynamic, mechanical, and dynamic stabilities of four crystal structures shown in Extended Data Figure 5. **a**, Formation enthalpies with respect to graphite as a function of applied pressure. Crystals containing gradia interfaces become stable energetically relative to graphite at high pressures in the range of 10−20 GPa. Note that Gradia-CO and Gradia-HB transform directly into CD and HD above 11 GPa and 19 GPa, respectively. This might be related to the high-energy *sp*^2^-hybridized atoms at gradia interfaces, which can contribute to diamond growth by advancing the interface towards the graphite side under pressure. **b**, Calculated elastic constants (*C*_ij_, GPa), bulk moduli (*B*, GPa), shear moduli (*G*, GPa), and Young's moduli (*E*, GPa) of hypothetical crystal structures at ambient pressure. Clearly, the calculated elastic constants *C*_ij_ of crystal structures satisfy the mechanical stability criteria, confirming their mechanical stability at ambient pressure. **c**, Calculated phonon spectra of the hypothetical crystal structures at ambient pressure. No imaginary phonon frequencies throughout the whole Brillouin zone indicates that all the structures are dynamically stable.

**Extended Data Fig. 8:**
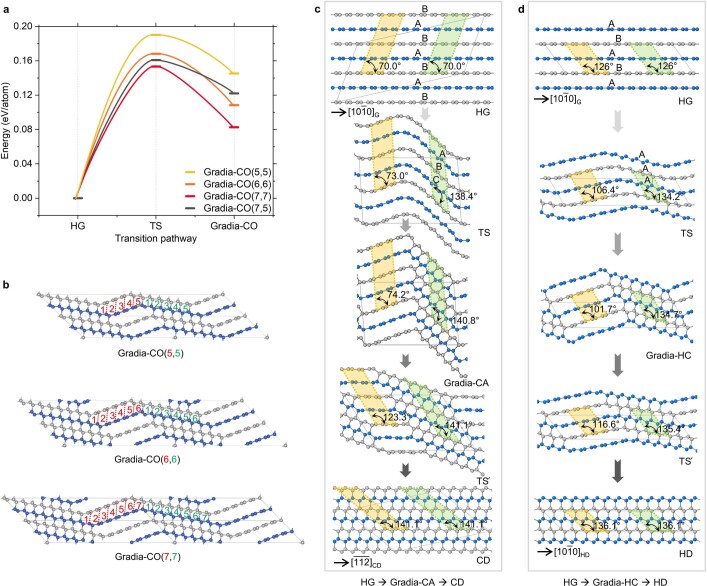
Transformation process from graphite to diamond through Gradia crystals. **a**, Energy profile of transformation from graphite to Gradia-CO structures with different unit cell size or different graphite and diamond fractions at 10 GPa. **b**, Nomenclature of Gradia-CO (*m*, *n*): *m* is the number of six-numbered rings to be formed between adjacent layers in graphite domain, and *n* is the number of six-numbered rings formed in diamond domain. Red dotted lines indicate further bonding to six-numbered rings for a complete transformed diamond. **c**, **d**, Structure snapshots during graphite-to-diamond transformation through Gradia-CA and Gradia-HC crystals, respectively, at 10 GPa.

To understand the origin of gradia interfaces and their roles in graphite-to-diamond transformation, we conducted first-principles calculations on intentionally designed hybrid crystals with the characteristic gradia interfaces shown in Fig. 3c (see Methods, Extended Data Figs. 5–7 and Extended Data Table 1 for more details). As shown in Extended Data Fig. 5, the unit cell for each hypothetic crystal is separated into *sp*^2^-hybridized graphitic (grey-coloured atoms) and *sp*^3^-hybridized diamond (gold-coloured atoms) sections that are bonded coherently through a gradia interface (green-coloured atoms). The thermodynamic, mechanical and dynamic stabilities of these crystal structures are shown in Extended Data Fig. 7. Transformation energy barriers from graphite to diamond through these intermediate crystal structures were evaluated under pressure with the variable-cell nudged-elastic-band (VCNEB) simulation method^29,30^ as implemented in the USPEX code^31,32^. The transformation processes are summarized in Fig. 4 and Extended Data Fig. 8. The energy barriers required to form gradia interfaces directly from graphite are all higher than those for diamond growth by advancing the gradia interfaces into graphite (Fig. 4a). It is noted that in all considered cases, the energy barriers decrease monotonically with increasing pressure in the range of 0–15 GPa (Fig. 4b,c), and the transformation barriers along the pathways through the gradia interfaces are substantially lower than those along classic concerted transformation pathways^29^. Moreover, the calculated transformation barrier from graphite to Gradia structures would decrease with increasing unit cell size or graphite fraction (Extended Data Fig. 8a,b).

Figure 4d,e and Extended Data Fig. 8c,d provide atomistic snapshots from pure graphite to diamond under 10 GPa through hypothetic crystals with different gradia interfaces (see Supplementary Videos 1–4 for the whole processes). During the transformation, graphite layers undergo wave-like bending with remarkable localized variations in stacking order and interlayer spacing, inducing additional bonding across adjacent graphite layers to form gradia interfaces in regions with suitable stacking order and interlayer spacing. For example, Fig. 4d shows five snapshots of the transformation from HG to CD. The oscillation of the graphitic layers (second snapshot from the top) results in localized changes of stacking order from AB to CBA accompanied by reduced interlayer spacing, leading to the formation of a Gradia-CO interface and the appearance of the first diamond-like bonding. At this diamond nucleation stage, the energy barrier reaches the maximum (Fig. 4a). The gradia interface then advances from both sides into graphitic sections, resulting in the growth of diamond lattice (third and fourth snapshots) until the transformation to CD is complete (fifth snapshot). In contrast, the previously proposed wave-like buckling and slipping mechanism invokes uniform interlayer distances without forming gradia interfaces^15^. Figure 4a suggests that once a gradia interface is formed, further formation of diamond is energetically favoured even under metastable conditions. This is confirmed by in situ STEM observations (Extended Data Fig. 3c,d). Under electron-beam irradiation in vacuum, new diamond-like atomic bonding is identified from the graphite side of the Gradia-CO interface. This remarkable observation is due to the lower energy barrier for diamond growth through step-by-step advancing of the gradia interface.

**Extended Data Fig. 9:**
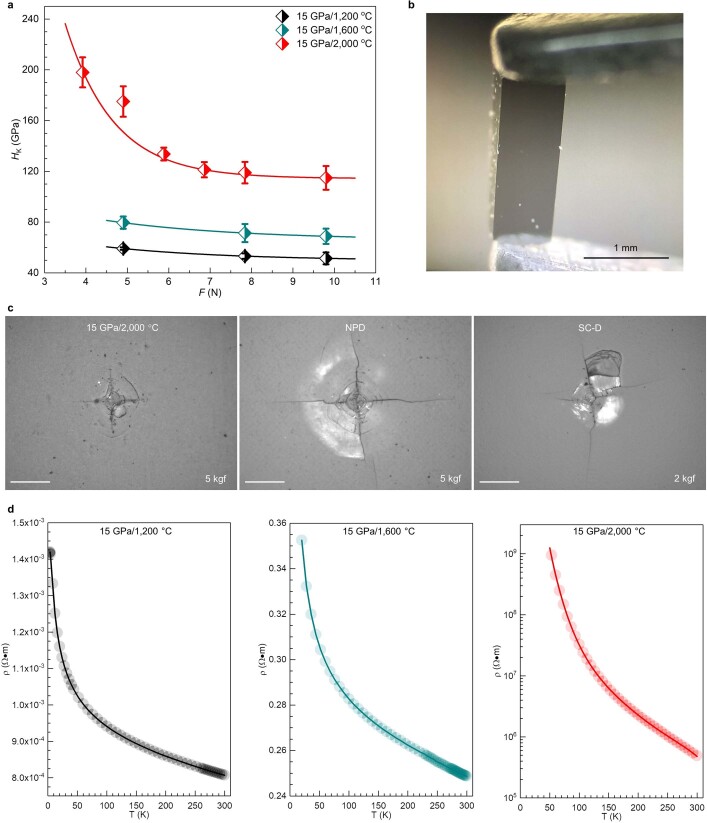
Mechanical and electrical properties of Gradia samples. **a**, Knoop hardness (*H*_K_) as a function of applied loads for Gradia samples quenched from 15 GPa/1,200 °C (black), 15 GPa/1,600 °C (turquoise), and 15 GPa/2,000 °C (red). Error bars represent one s.d. (*n* = 5). *H*_K_ of three samples under 9.8 N load are 51 ± 4.7, 69 ± 6.0, and 115 ± 9.3 GPa, respectively. In comparison, *H*_K_ of binderless nanopolycrystalline diamond (NPD) under 9.8 N is 111 ± 15.2 GPa. **b**, A photograph of black Gradia sample with a polished surface (15 GPa/2,000 °C). **c**, Vickers indentation fracture toughness of Gradia (15 GPa/2,000 °C), binderless nanopolycrystalline diamond (NPD) and single-crystal diamond (SC-D, (111) face)_._ The estimation of fracture toughness of materials is based on the length of cracks generated on the sample with a Vickers indenter of square-pyramid diamond under high loads of 49 N (for Gradia and NPD) and 19.6 N (for SC-D). All scale bars are 20 µm. Unlike other materials with obvious cracks, no visible crack was generated in Gradia, indicating an excellent toughness of Gradia. The fracture toughness is 8.7 ± 1.8 MPa·m^0.5^ for NPD and 6.4 ± 1.1 MPa·m^0.5^ for SC-D. **d**, Temperature-dependent electrical resistivities of Gradia samples, showing a semiconducting characteristic. With increasing synthesis temperature, the diamond content in Gradia increases gradually, resulting in an increase in electrical resistivity. Gradia samples recovered from 15 GPa/1,200 °C and 15 GPa/1,600 °C show low resistivities, while the sample recovered from 15 GPa/2,000 °C shows a significantly high resistivity. The mechanical and electrical properties of Gradia can be tunable by adjusting the proportions of graphite and diamond in the bulk.

By integrating *sp*^2^-hybridized graphite and *sp*^3^-hybridized diamond nanodomains with strong coherent interfaces, Gradia has the prospect of combining the advantages of both parties, with potentially a wide range of properties for multifunctional applications^4^. The gradia interfaces may also play a substantial role in tuning material properties. For example, the calculation results suggest that the designed hybrid crystals display obvious metallicity (Extended Data Figs. 5 and 6), contributed mostly by atoms in the graphitic section and gradia interface. It is noted that the contribution to the metallicity from interface atoms is comparable to, or even higher than, that from graphitic atoms in Gradia-CO and Gradia-HB crystals, owing to the presence of *sp*^2^-hybridized atoms (circled in red) at the interface. In Gradia, the proportion-tunable graphite and diamond domains together with the versatile gradia interfaces offer additional freedom in engineering nanostructures, for desired properties. Specifically, differently hybridized carbon atoms in Gradia contribute to different functionalities, for example, *sp*^3^ atoms to superhardness, *sp*^2^ atoms to electrical conductivity, and *sp*^2^–*sp*^3^ mixed atoms near the interfaces to toughness^4^. With regulated fractions and distributions of different types of atom, a variety of properties, which are inaccessible for diamond and graphite separately, may be tailored for Gradia (Extended Data Fig. 9).

The transformation from graphite to diamond under static compression occurs in two stages, that is, the formation of a coherent gradia interface (diamond nucleation) and subsequently the advance of the interface (diamond growth). The transformation mechanism clarified in this work can serve as guidance in understanding the transformations of boron nitride and other carbon phases such as carbon nanotubes and onions under high pressure. Beyond the transformation mechanism, the observed Gradia marks a major step towards nanostructure and properties engineering in diamond-related materials, and provides opportunities in pursuing desired combination of mechanical and electronic properties, such as simultaneous superhardness, high toughness and electrical conductivity.

## Methods

### Sample synthesis

The Gradia-containing samples were synthesized from graphite (99.99%, Alfa Aesar) under conditions of 10–25 GPa and 1,000–2,300 °C. HPHT experiments were performed with a 10-MN double-stage large-volume multi-anvil system by using standard COMPRES 10/5 (or 8/3) sample assembly consisting of a 10-mm (or 8-mm) spinel + magnesium oxide octahedron with a rhenium heater and a LaCrO_3_ thermal insulator. Temperature was measured with type-C tungsten–rhenium thermocouples, and pressure was estimated from previously determined calibration curves. During the synthesis, pressure increased at 2 GPa per hour to the target pressure; then the sample was heated at a rate of 20 °C per minute to the target temperature. In all the experiments, the sample was maintained under the target pressure and temperature for 2 h. After that, the sample was cooled to room temperature at 50 °C per minute, followed by pressure release at a rate of 2 GPa per hour. Back-transformation of diamond during pressure release at room temperature is unlikely, considering that graphitization of diamond occurs only at high temperature. The recovered sample rods were 1–2.5 mm in diameter and height.

### Ultra-thin TEM sample preparation

To eliminate grain overlaps in STEM imaging, foils with a thickness of about 60 nm were cut with a focus ion beam (FEI Helios 5 CX DualBeam), and further thinned to 20 nm with low-energy argon-ion milling (Fischione Model 1040 NanoMill). Before loading into the microscope, the foils were cleaned with H_2_/O_2_ plasma (Gatan 695 Plasma cleaner) for 40 s to eliminate possible carbon contamination.

### HAADF-STEM measurement

STEM measurements were conducted with a spherical aberration-corrected scanning transmission electron microscope (FEI Themis Z), with a monochromator, operating at an accelerating voltage of 300 kV. The electron-beam damage to the STEM specimen was consciously avoided or minimized in the STEM observations. In low-magnification BF-STEM mode, the electron irradiation dose is relatively low, which cannot cause noticeable damage to the sample. For high-resolution STEM observations, a very low beam current of 50 pA was used to reduce irradiation damage, with a short dwelling time of 0.2 μs. BF, low-angle ADF (LAADF) and HAADF images were obtained by combining 20 frames from acquired series with drift correction (DCFI in software of Velox, Thermo Fisher). No obvious change in the interface structure was found by comparing these frames, indicating that electron irradiation damage on the interface structure is negligible. The probe convergence angle was set at 25 mrad. The collecting angles of BF and LAADF were set at 6 mrad and 16−62 mrad, respectively. The collecting angle of HAADF was set at 65−200 mrad to eliminate effects of coherent scattering.

### X-ray diffraction

Powder X-ray diffraction patterns of recovered samples were collected using Bruker D8 Discover (Cu Kα).

### Mechanical property measurement

A microhardness tester (KB 5 BVZ) was used to measure Vickers hardness *H*_V_ and fracture toughness *K*_Ic_ of samples by using a diamond Vickers indenter, and to measure Knoop hardness *H*_K_ with a diamond Knoop indenter. *H*_V_ was determined from *H*_V_ = 1,854.4*P*/*d*_1_^2^, where *d*_1_ (µm) is the arithmetic mean of the two diagonals of Vickers indentation. *H*_K_ was determined from *H*_K_ = 14,229*P*/*d*_2_^2^, where *P* (N) is the applied load and *d*_2_ (µm) is the major diagonal length (long axis) of rhomboid-shaped Knoop indentation. The adopted loading and dwelling times were 40 s and 20 s, respectively. Five hardness data points were obtained at each load, and the hardness values were determined from the asymptotic-hardness region. *K*_Ic_ was calculated from *K*_Ic_ = 0.016(*E*/*H*_V_)^0.5^*F*/*C*^1.5^ for radial cracks formed on surfaces of bulk samples, where *F* (in N) is the applied load, *C* (in µm) is the average length of the radial cracks measured from the indent centre, and *E* is Young’s modulus, which is 1,140 GPa for diamond.

### Electrical resistivity measurement

The electrical resistivities of samples were measured in the range of 4–300 K by using the van der Pauw method in the Physical Property Measurement System (PPMS, Quantum Design). The effects of electrodes on the resistivity measurements can be avoided through transforming the current direction in different van der Pauw probes. Four electrodes were taped onto insulating quartz plates (5 × 5 mm in size) and placed onto the sample for conductivity measurements. The sample surfaces were first polished with diamond submicrometre powder before measurement.

### Density-functional-theory simulation

The hypothetic crystal structures based on gradia interfaces were constructed with the Materials Visualizer module in Materials Studio (Accelrys Software). The calculations were performed on the basis of density functional theory as implemented in the CASTEP code^33^, and the ultrasoft pseudopotentials were used^34,35^. The local density approximation exchange-correlation functional of Ceperley and Alder parameterized by Perdew and Zunger (CA–PZ) was utilized for structural optimization and calculations of total energies, band structures, elastic properties and phonon spectra^36,37^. A *k*-point sampling^38^ of 2π × 0.03 Å^−1^ and a plane-wave cut-off of 600 eV were used. Band structures were also calculated with Perdew−Burke−Ernzerhof (PBE) and Heyd–Scuseria–Ernzerhof (HSE06) functionals as implemented in the Vienna ab initio simulation package (VASP)^39^ to illustrate the reliability of the band structure calculations (Extended Data Fig. 6). The selected calculation parameters were all tested to ensure that energy convergence was less than 1 meV per atom. For comparison, we also performed similar calculations on pure graphite and diamond crystals. To reveal the transformation mechanism from graphite to diamond through the gradia interfaces, we performed VCNEB simulations^29,30^ at 0 GPa, 5 GPa, 10 GPa and 15 GPa, as implemented in the USPEX code^31,32^. Both initial and final states were relaxed at set pressures. Then, the initial pathways were subsequently refined by the VCNEB method and optimized to find the minimum-energy pathways. The forces and stresses were computed by VASP code^39^ with the local density approximation exchange-correlation functional of CA–PZ^36,37^. The projector augmented-wave method was adopted, with 2*s*^2^2*p*^2^ treated as valence electrons for the C atom. Plane-wave cut-off energies were 600 eV and *k*-point meshes were sampled with the resolution of 2π × 0.04 Å^−1^. Spring constants were set as 40 eV Å^−2^. The climbing image technique^40^ was used to precisely locate transition states after hundreds of VCNEB steps. For the mechanical stability, the 13 independent elastic constants *C*_*ij*_ for a stable monoclinic structure should satisfy the Born stability criteria^41–44^.

## Online content

Any methods, additional references, Nature Research reporting summaries, source data, extended data, supplementary information, acknowledgements, peer review information; details of author contributions and competing interests; and statements of data and code availability are available at 10.1038/s41586-022-04863-2.

### Supplementary information

Supplementary Video 1 Transformation process from graphite to cubic diamond through Gradia-CO at 10 GPa. The detailed analysis is shown in Fig. 4.

Supplementary Video 2 Transformation process from graphite to cubic diamond through Gradia-CA at 10 GPa. The detailed analysis is shown in Fig. 4 and Extended Data Fig. 8c.

Supplementary Video 3 Transformation process from graphite to hexagonal diamond through Gradia-HB at 10 GPa. The detailed analysis is shown in Fig. 4.

Supplementary Video 4 Transformation process from graphite to hexagonal diamond through Gradia-HC at 10 GPa. The detailed analysis is shown in Fig. 4 and Extended Data Fig. 8d.

## Figures and Tables

**Extended Data Table 1:**
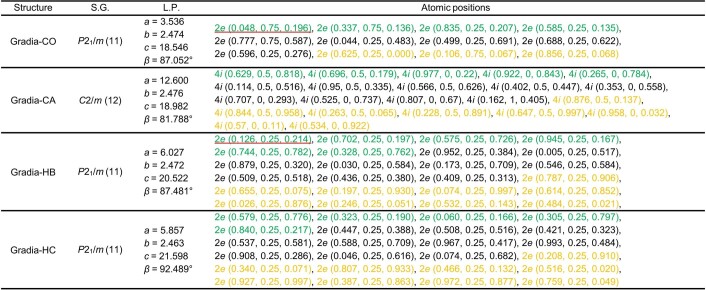
Space group (S.G.), lattice parameters (L.P., Å) and atomic Wyckoff positions of the hypothetical Gradia crystal structures shown in Extended Data Fig. 5 at ambient pressure

The interface, graphitic and diamond-structured atoms are coloured in green, grey and gold, respectively. Among the interface atoms, the underlined atoms are *sp*^2^-hybridized, the others are *sp*^3^-hybridized.

## Data Availability

The data that support the findings of this study are available from the corresponding author upon request.
